# mPUMA: a computational approach to microbiota analysis by *de novo* assembly of operational taxonomic units based on protein-coding barcode sequences

**DOI:** 10.1186/2049-2618-1-23

**Published:** 2013-08-15

**Authors:** Matthew G Links, Bonnie Chaban, Sean M Hemmingsen, Kevin Muirhead, Janet E Hill

**Affiliations:** 1 Agriculture and AgriFood Canada, 107 Science Place, S7N 0X2, Saskatoon, SK, Canada; 2 Department of Veterinary Microbiology, University of Saskatchewan, 52 Campus Drive, S7N 5B4, Saskatoon, SK, Canada; 3 National Research Council Canada, 110 Gymnasium Place, S7N 0W9, Saskatoon, SK, Canada; 4 Department of Microbiology & Immunology, University of Saskatchewan, 107 Wiggins Road, S7N 5E5, Saskatoon, SK, Canada

**Keywords:** Operational taxonomic unit, Assembly, Automated sequence analysis pipeline, 60 kDa chaperonin, *Cpn*60, Barcode, Microbial profiling, Microbiota, Microbiota analysis

## Abstract

**Background:**

Formation of operational taxonomic units (OTU) is a common approach to data aggregation in microbial ecology studies based on amplification and sequencing of individual gene targets. The *de novo* assembly of OTU sequences has been recently demonstrated as an alternative to widely used clustering methods, providing robust information from experimental data alone, without any reliance on an external reference database.

**Results:**

Here we introduce mPUMA (microbial Profiling Using Metagenomic Assembly, http://mpuma.sourceforge.net), a software package for identification and analysis of protein-coding barcode sequence data. It was developed originally for *Cpn*60 universal target sequences (also known as *Gro*EL or *Hsp*60). Using an unattended process that is independent of external reference sequences, mPUMA forms OTUs by DNA sequence assembly and is capable of tracking OTU abundance. mPUMA processes microbial profiles both in terms of the direct DNA sequence as well as in the translated amino acid sequence for protein coding barcodes. By forming OTUs and calculating abundance through an assembly approach, mPUMA is capable of generating inputs for several popular microbiota analysis tools. Using SFF data from sequencing of a synthetic community of *Cpn*60 sequences derived from the human vaginal microbiome, we demonstrate that mPUMA can faithfully reconstruct all expected OTU sequences and produce compositional profiles consistent with actual community structure.

**Conclusions:**

mPUMA enables analysis of microbial communities while empowering the discovery of novel organisms through OTU assembly.

## Background

A common approach to the profiling of complex microbial communities is the amplification and sequencing of ‘universal’ genes, such as *Cpn*60 (also known as *Gro*EL or *Hsp*60) or *16S rRNA*, as DNA barcodes for the genomes in which they reside. Barcodes are defined by the International Barcode of Life Project as short, phylogenetically informative sequences from standardized regions of the genome that can be used for species identification and discovery [1], and preferred barcodes for microbes including fungi [2] and bacteria [3] have been proposed recently. In microbial community studies, broad-range ‘universal’ PCR primers are used to amplify regions of the target genes, and amplicon sequences are determined directly using next-generation sequencing methods. These gene-targeted methods arguably fall under the umbrella of 'metagenomics' along with whole genome sequencing approaches, since these are methods based on the analysis of total genomic content of a community of organisms rather than individual isolates [4]. The number of individual sequences generated is typically in the order of 10^6^ and can be much greater. Thus, some form of data aggregation is required to reduce the complexity of the raw sequence data, and facilitate interpretation. Data aggregation is focused on the *in silico* steps following sequence data acquisition, and not issues that arise from methods of DNA extraction and possible biases in PCR amplification. The key challenge in aggregation is ensuring that the resulting 'profiles' (list of sequences and their abundances) are faithful to the raw sequence data that was aggregated.

Currently, the most widely used method for data aggregation is the formation of operational taxonomic units (OTU) with clustering approaches such as those of MOTHUR [5] or UCLUST [6] as implemented within packages such as QIIME [7]. Clustering procedures culminate in the selection of a representative sequence for each OTU, which may be selected from the experimental data according to various rules: longest sequence in the cluster, most abundant sequence in the cluster, or random selection. However, representative sequences selected from the experimental data may not include full-length coverage of the target, depending on its length. This in turn limits information content, and the ability to conduct multiple sequence alignments and phylogenetic analysis for characterization of novel OTU sequences. Alternatively, the closest sequence from a reference database may be used to represent the OTU [5]. A limitation common to all of these approaches is apparent when the community under study contains novel sequences not represented in reference databases. In these cases, novel sequences in the experimental data may be ignored or pooled together as 'unclassified' since they do not closely resemble the reference sequences. The end result is that the aggregated description of the community may not reflect the input sequence data generated in the experiment.

We have demonstrated recently that *de novo* assembly of OTU sequences is an alternative strategy for sequence data aggregation that provides robust information from experimental data alone [3]. In this approach, OTU sequences are consensus sequences derived from the experimental data, without any reliance on an external reference database. This strategy has been used successfully in producing high resolution profiles of a variety of complex microbial communities [8-10] and has led to the resolution of subspecies level diversity within previous established bacterial 'species' [11]. However, until now there has been no computational pipeline available for this work, requiring practitioners to attend to each step of the assembly and post-assembly analysis individually. Here, we introduce mPUMA (microbial profiling using metagenomic assembly), a computational pipeline for the automated assembly and analysis of OTU sequences from protein coding gene sequence data derived from microbial communities.

## Methods

### mPUMA workflow

**Figure 1:**
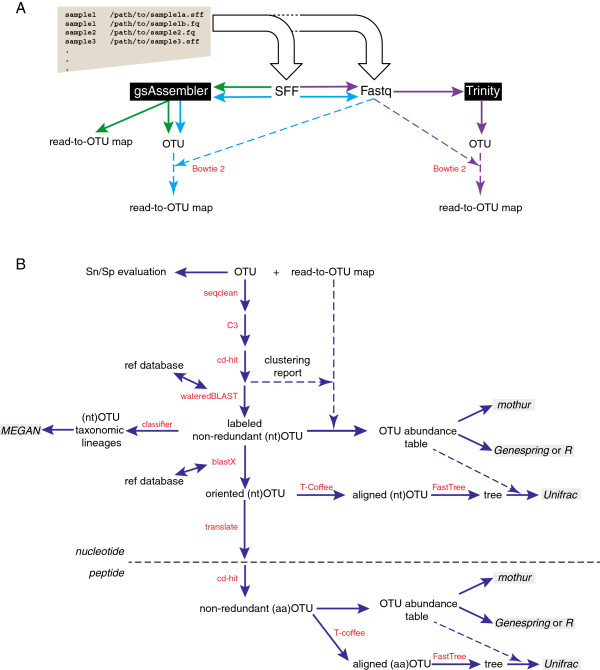
**mPUMA workflow.** Programs used at each step in the pipeline are shown in red. **A**. User-defined protocol options for assembly and read-to-operational taxonomic unit (OTU) tracking include gsAssembler for both processes (green arrows), gsAssembler plus Bowtie 2 for read tracking (blue arrows), and Trinity assembly plus Bowtie 2 for read tracking (purple arrows). **B**. Post-assembly analysis of OTU and abundance data. Gray boxes indicate possible downstream analysis tools for which input is generated by mPUMA. The horizontal broken line indicates the transition from analysis of nucleotide OTU ((nt)OTU) and translated peptide OTU ((aa)OTU). Quality of the assembly can be evaluated by assessing Sensitivity/Specificity (Sn/Sp) of each OTU as defined in [3]. WateredBLAST is a combination of BLAST and Smith-Waterman alignments, described in detail in [15].

mPUMA was written in PERL using BioPerl [12] and is maintained as a sourceforge project (http://mpuma.sourceforge.net/). It was developed originally for assembly of *Cpn*60 universal target sequences [13,14] since the characteristics of this target make it a preferred sequence barcode for resolution of bacterial taxa [3]. However, mPUMA is applicable to any other suitable molecular barcode. mPUMA assembles OTU from PCR amplicon sequence libraries generated from any number of samples, starting from a set of SFF or Fastq files, and a text file explaining how the files relate to experimental samples. Following assembly, the abundance of each OTU is determined and files for downstream analysis using several common microbial ecology and phylogeny tools are generated. The mPUMA workflow is illustrated in Figure 1.

### Sequence assembly

Sequence assembly within mPUMA can be performed by two methods: gsAssembler (Roche/454, Branford, CT, USA) in cDNA mode, or Trinity [16]. Abundance per OTU can be calculated by mPUMA from a read-to-OTU map produced in one of two ways (Figure 1A). For gsAssembler assemblies, the internal read tracking of the assembly process can be used as the basis for the read tracking. Alternatively, reference mapping with Bowtie 2 [17] can be used to map each experimental read onto reference OTUs assembled with either gsAssembler or Trinity. Considerations for the optimal assembly and read tracking strategy for any particular project are discussed below. Regardless of the strategy used, the quality of the assembly and read tracking result is assessed in terms of the specificity and sensitivity of each OTU as described previously [3].

### Post-assembly analysis of OTU

Removal of PCR primer sequences is accomplished with seqclean (http://sourceforge.net/projects/seqclean/files/). Identification and removal of chimeric sequences is performed by two strategies implemented within mPUMA. First, gsAssembler identifies chimeras resulting from the assembly process. Second, the Chaban Chimera Checker (C3) identifies putative chimeras that may be removed from subsequent analyses. In C3 the 5′ and 3′ ends of each OTU (150 bp) are extracted, compared to a reference set of sequences (for example, a non-redundant set of sequences from cpnDB [14]) and evaluated to see if both ends match the same reference sequence in the expected orientations. Putative chimeras are identified as assembled OTU that fail this test. In novel environments where taxa are not well represented in the reference database, it may be appropriate to forego the use of C3 because the novelty of the experimental sequences could lead to an increased false positive rate in chimera identification.

Non-chimeric OTU are clustered at 100% identity by CD-hit [18] to remove redundant sequences. For protein coding barcode sequences, mPUMA implements BLASTX [19] to identify the correct reading frame for translation of OTU, and then translates the nucleotide OTU to their corresponding peptide OTU sequences. Redundant peptide sequences are also collapsed using CD-hit [18] at 100% identity. mPUMA calculates the abundance of each non-redundant peptide OTU for each library, resulting in a peptide OTU abundance table.

Nucleotide and peptide OTU and abundance data are formatted for use with additional tools, which are run automatically where appropriate. Prior to generating input files for these applications, mPUMA carries out a down-sampling process where reads are sampled at random to the depth of the smallest library to address the concerns raised by Gihring *et al*. related to the effects of unequal sampling effort on calculation and comparison of ecological parameters such as richness, diversity and evenness [20]. Abundance files for OTU are used to create input for MOTHUR [5]. Using t-coffee [21] for multiple sequence alignments and FastTree [22], a phylogenetic tree of the OTU is calculated, which can be used in conjunction with abundance data to analyze libraries in Unifrac [23,24]. A naïve Bayesian classifier trained on *Cpn*60 universal target sequences from cpnDB [14] has been developed using the RDP classifier framework [25]. Classifier results can be loaded into MEGAN [26] for comparison of multiple libraries in a taxonomic context. All of the output files generated by mPUMA for secondary analyses are generated both for the nucleotide and the amino acid OTU sequences.

### Computational platform

Demonstrations of mPUMA running in an unattended fashion were performed using a previously published dataset [10] that included 711 MB of data in SFF files. Analyses were carried out on a Dell R910 equipped with 128 GB of RAM and 2x Intel Xeon 6-core E7530 processors running CentOS 5.8.

## Results and Discussion

**Figure 2:**
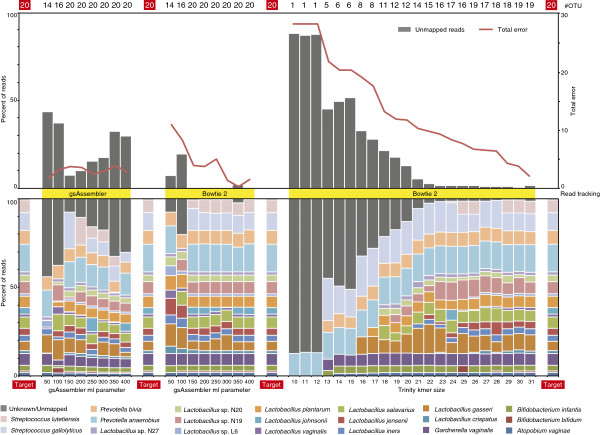
**Comparison of methods for both assembly and abundance calculation using a synthetic community of 20 cloned *Cpn*60 universal target sequences.** Three different scenarios were investigated for the generation of a microbial profile (left-to-right): gsAssembler alone, gsAssembler plus Bowtie 2 for abundance, and Trinity plus Bowtie 2 for abundance. The number of community members recovered is shown across the top (out of 20). The major parameter affecting the accuracy of assembly is varied across the lower x-axis. For gsAssembler the minimum identity of overlaps was held constant at 90 while the minimum length parameter was varied. In the case of Trinity, the k-mer length was varied from 10 to 31 bp. The upper panel shows the percentage of reads which were untrackable (left ordinate) and the total error associated with each assembly (right ordinate). In the lower panel, microbial profiles are plotted as stacked bars with each element colored by organism according to the legend. Profiles marked as 'Target' indicate the actual composition of the amplicon library determined by Bowtie 2 mapping of all reads on to the 20 reference sequences.

To validate the primary function of mPUMA (OTU formation and abundance calculation), we tested its performance in the analysis of sequence data generated by amplification and sequencing of *Cpn*60 universal target sequences from a synthetic community containing cloned *Cpn*60 universal target sequences from 20 human vaginal bacteria with pairwise sequence identity values of 60 to 96% [27]. PCR from this template mixture and pyrosequencing of the resulting amplicon library on a Roche GS FLX instrument was performed using established protocols [28], resulting in 9,877 sequence reads from either the 5′ or 3′ end of the target sequence. The SFF data is accessible through the mPUMA sourceforge site (http://mpuma.sourceforge.net/). We verified that all 20 target sequences were represented in the results by using Bowtie 2 to map all reads on to the reference sequences for the synthetic community ('Target' in Figure 2). OTU formation and abundance calculations were performed on the dataset using all three options available within the mPUMA pipeline (gsAssembler OTU assembly/gsAssembler read-to-OTU mapping, gsAssembler OTU assembly/Bowtie 2 read-to-OTU mapping and Trinity OTU assembly/Bowtie 2 read-to-OTU mapping) and the resulting microbial profiles were evaluated for number of OTU generated, number of reads unmapped, amount of total error generated and comparison of the profile to the known 'Target' synthetic community profile (Figure 2).

gsAssembler was able to reconstruct all 20 expected OTU with minimum length parameter settings of >100 bp (Figure 2). However, despite accurately describing the richness of the sample (20 OTUs), read tracking within gsAssembler failed to place a substantial proportion of data in any OTU. The proportion of sequence reads unmapped increased steadily from 8% to a maximum of 33% as the minimum length parameter was increased from 150 through 350 bp (Figure 2). There are several possible explanations for this unplaced data: the reads could be short or of low quality, or the assembly process may not have completely accounted for the placement of each read to an OTU. In our experience, situations in which a study contains samples with extreme differences in richness can lead to incomplete mapping when utilizing gsAssembler which cannot be resolved using the available command line options (−ig, -it, and -icc). The occurrence of such 'thresholding' problems is recorded in the 454IsotigsLayout.txt files generated by gsAssembler. Given that we confirmed that gsAssembler had correctly resolved all 20 of the expected OTU for this synthetic community, we were left with the possibility that either there was a proportion of the data which was of insufficient quality and/or length to be placed in the OTUs at higher stringencies (that is, greater minimum overlap length requirement) or the placement was incomplete. To determine which of these phenomena were occurring we employed Bowtie 2 [17] as a method to independently assess the read to OTU mapping.

When read mapping was performed using Bowtie 2 to place reads onto a gsAssembler assembly, there was a dramatic reduction in the proportion of unmapped data and in total error of the assembly coincident with all 20 members of the synthetic community being resolved (Figure 2). The results of assembly using gsAssembler with a minimum overlap >100 bp followed by read mapping with Bowtie 2 served to construct a microbial profile indistinguishable from the actual profile of the synthetic community at both the nucleotide and peptide levels, with the 20 expected nucleotide OTU and 19 corresponding peptide OTU (peptide sequences for *Lactobacillus gasseri* and *Lactobacillus johnsonii* are identical). This result confirmed that the reads were of sufficient length and quality for inclusion, and thus the more likely explanation for the relatively large proportion of data that is not placed by gsAssembler read tracking is that the assembler had failed to completely assign all reads to the OTU assembled (the thresholding problem described above).

gsAssembler uses an Overlap-Layout-Consensus (OLC) strategy for assembly, which is dramatically affected by coverage depth [29]. The dominant alternative approach for assembly is the use of a de Bruijn graph (DBG) to analyze sequence composition in terms of k-mers. The total length of sequence being assembled, independent of coverage depth, governs the size of a de Bruijn graph. Being unaffected by coverage depth is the chief computational advantage of DBG approaches. We explored whether Trinity, a DBG method [16], offers a valid alternative to gsAssembler in cDNA mode for the analysis of microbial barcode data. Within Trinity, the parameter most likely to affect the accuracy of assembly results is k-mer size. We examined all possible k-mer lengths supported by Trinity (k-mer ranging from 10 to 31, inclusive). Bowtie 2 was then used to map the individual reads onto the non-redundant set of OTU formed by Trinity for calculating abundance because the reductive process of distilling sequences to component k-mers eliminates the ability of tracking reads directly within DBG approaches.

As can be seen in Figure 2, increasing k-mer length resulted in the formation of more of the expected OTU, reduction of the proportion of unmapped reads and a corresponding reduction in total error of the assembly. However, in no case did Trinity resolve all 20 OTUs from the synthetic community. Trinity assemblies with a k-mer of 30 or 31 were nearly complete, failing only to resolve an OTU for *L. johnsonii*. This was perhaps not surprising since *L. johnsonii* and *L. gasseri* are the two most similar members of the community (96% identical) and have similar abundances, being the 11th and 9th most abundant in this dataset, respectively. The *L. johnsonii* reads were placed in the *L. gasseri* OTU when an *L. johnsonii* OTU was not formed.

Resource usage by mPUMA can vary significantly depending on the size and complexity of the datasets being analyzed. In our experience the use of Trinity over gsAssembler can be necessary for computational constraints (memory and cpu time) when dealing with datasets that are extremely rich or diverse. mPUMA is suitable for the assembly and analysis of OTU from other suitable targets besides *Cpn*60, such as the gene encoding the universal archaeal type-II chaperone (also known as Thermosome or TCP1 or CCT) [30], and *Rpo*B [31]. Pyrosequencing data from both have been processed through mPUMA, confirming its utility for other protein coding targets. To date, we have applied mPUMA to the analysis of amplicon sequence data from the 454 GS FLX, Titanium and Junior platforms. We encourage the microbial ecology community to investigate the application of mPUMA to other sequence data types and gene targets of interest.

## Conclusions

The *de novo* assembly of OTUs from barcode sequence data can be optimized to reduce error and accurately reflect the richness of a microbial community, presenting possible advantages over clustering methods that may mask diversity or inhibit discovery of novel sequences. The mPUMA pipeline was developed to facilitate the use of assembly in microbial ecology studies where both accurate descriptions of richness and calculation of OTU abundance are desired. Based on our examination of a synthetic community, optimal resolution of OTU sequence barcodes and calculation of their abundance can be achieved through use of gsAssembler with a minimum overlap length parameter >100 bp followed by Bowtie 2 read tracking for determining OTU abundance. In cases where computational performance is limiting, Trinity assembly followed by read tracking with Bowtie 2 should produce near-optimal results with only exceptionally similar barcodes remaining unresolved. In choosing the most appropriate strategy for assembly and abundance calculations from among the options available in mPUMA, researchers will need to balance the computational performance of the assembly approach with the precision of OTU formation.

The mPUMA software package is available from sourceforge and it is covered by an open-source license (http://mPUMA.sourceforge.net). At present, mPUMA is distributed on its own, but it is possible that in the future it may become incorporated into a Virtual Machine image. Since it is as an open-source platform, mPUMA can be extended by anyone interested in utilizing *de novo* assembly for the analysis of microbial profiling data.

## Availability of supporting data

The SFF data used in the validation and demonstration of mPUMA is available through the mPUMA sourceforge site (http://mpuma.sourceforge.net/).

## Abbreviations

DBG: De Bruijn graph; mPUMA: microbial Profiling Using Metagenomic Assembly; OLC: Overlap-layout-consensus; OTUs: Operational taxonomic units.

## Competing interests

The authors declare that they have no competing interests.

## Authors’ contributions

MGL designed mPUMA. MGL and KM developed the mPUMA codebase. JEH, BC and SMH contributed to the design and validation of mPUMA, and data analysis. BC designed the C3 chimera checker and generated the *Cpn*60 amplicon library. MGL and JEH drafted the manuscript and figures. All authors read and approved the final manuscript.
